# The Evolving Landscape of Biomarkers in Celiac Disease: Leading the Way to Clinical Development

**DOI:** 10.3389/fimmu.2021.665756

**Published:** 2021-04-07

**Authors:** Glennda Smithson, Jenifer Siegelman, Toshihiko Oki, Joseph R. Maxwell, Daniel A. Leffler

**Affiliations:** ^1^ Research and Development, Takeda Pharmaceuticals Inc. Co., Cambridge, MA, United States; ^2^ Celiac Disease Research Program, Harvard Medical School, Boston, MA, United States

**Keywords:** celiac disease, biomarkers, patient populations, diagnosis, disease monitoring, clinical development

## Abstract

Celiac disease is a common immune-mediated disease characterized by abnormal T-cell responses to gluten. For many patients, symptoms and intestinal damage can be controlled by a gluten-free diet, but, for some, this approach is not enough, and celiac disease progresses, with serious medical consequences. Multiple therapies are now under development, increasing the need for biomarkers that allow identification of specific patient populations and monitoring of therapeutic activity and durability. The advantage of identifying biomarkers in celiac disease is that the underlying pathways driving disease are well characterized and the histological, cellular, and serological changes with gluten response have been defined in gluten challenge studies. However, there is room for improvement. Biomarkers that measure histological changes require duodenal biopsies and are invasive. Less invasive peripheral blood cell and cytokine biomarkers are transient and dependent upon gluten challenge. Here, we discuss established biomarkers and new approaches for biomarkers that may overcome current limitations.

## Introduction

Celiac disease (CeD) is a chronic disease mediated by a destructive immune response triggered by gliadin, a protein found in wheat, rye, and barley. The response to gliadin is characterized by activation of gliadin-specific T cells, anti-gliadin and tissue transglutaminase antibody response, and small intestine inflammation and damage to the epithelium resulting in a characteristic villous flattening (1). Biopsy-confirmed CeD currently has a worldwide prevalence of 0.7% and has been increasing in prevalence over the last 3 decades (2).

In most patients, eliminating gluten from the diet (gluten-free diet; GFD) reduces symptoms and recurring intestinal damage. However, for about 30% of patients, gluten restriction is not sufficient to prevent symptoms or damage (3, 4). This lack of response is most commonly due to repeated inadvertent gluten exposure and/or high sensitivity to gluten at levels below what is considered ‘gluten free’ [20 ppm; (5)], but, in rare cases, may be related to refractory celiac disease (RCD). A diagnosis of RCD is based on continued intestinal damage and malabsorption after ≥ 12 months on a GFD. RCD1 is similar to active CeD with CD3+ polyclonal T cells comprising the majority of intraepithelial lymphocytes. Often, this disease type improves over time with strict adherence to a GFD. RCD2, in contrast, is characterized by the clonal expansion of aberrant intraepithelial lymphocytes that do not express surface CD3 or a T-cell receptor. These patients have a much poorer prognosis, with higher mortality and likelihood of progressing to enteropathy-associated T-cell lymphoma (3, 6–8).

There are currently no approved therapies for CeD; however, there are several therapies in development. Larazotide acetate (INN-202) is the most advanced program and is currently in phase III (ClinicalTrials.gov identifier: NCT03569007). Phase I/II programs include TAK-062 (ClinicalTrials.gov identifier: NCT03701555) and latiglutenase (IMGX003), which degrade ingested gliadin (9)*;* PRV-015 (AMG 714), a monoclonal antibody that blocks IL-15, a cytokine associated with mucosal damage (10); and TAK-101, which elicits gliadin-specific immune tolerance. These therapies may target patients who are on a GFD but have ongoing symptoms and/or intestinal damage due to inadvertent gluten exposure. Currently, a diverse range of mechanisms is being investigated (11), and it is possible that selected therapeutics could be used for a broader segment of the patient population.

As the number of promising therapies for CeD grows, so does the need to measure therapeutic impact on clinically relevant endpoints and distinguish between different patient populations. This could be addressed, at least in part, by thoughtful biomarker selection. The Biomarkers, EndpointS and other Tools resource glossary (12) defines a biomarker as “*A defined characteristic that is measured as an indicator of normal biological processes, pathogenic processes, or responses to an exposure or intervention, including therapeutic interventions. Molecular, histologic, radiographic, or physiologic characteristics are types of biomarkers. A biomarker is not an assessment of how a patient feels, functions, or survives*”. In this review, we describe the context of use and limitations of current biomarkers, and how new biomarkers under development may avoid these limitations and play an important part in the clinical development of new therapies.

## Prognostic Biomarkers and Predicting the Course of CeD

Prognostic biomarkers provide information about the likelihood of a clinical event, disease recurrence, or disease progression within a patient population (12). For example, a prognostic biomarker in CeD might predict if a patient had a greater likelihood of a negative response to gluten exposure, or was more likely to progress to a serious disease state such as RCD. Some, but not all, prognostic biomarkers are also predictive biomarkers, allowing the selection of patients who are more likely to have a favorable/unfavorable response to a specific therapy.

Human leukocyte antigen (HLA) class II has been proposed to be a prognostic genetic biomarker for CeD severity (**Table 1**) (9, 10, 13–30). Either HLA-DQ2.5 (encoded by the HLA-DQA allele, HLA-DQA1*05 and HLA-DQB allele HLA-DQB1*02), HLA-DQ2.2 (HLA-DQA1*02 and HLA-DQB1*02 alleles), or HLA-DQ8 (HLA-DQA1*03 and HLA-DQB1*03:02 alleles) are present in almost all patients with CeD (31). HLA-DQ2 and/or HLA-DQ8, expressed on antigen-presenting cells, bind to immunogenic gliadin peptides and activate gliadin-specific CD4+ effector T cells (1). HLA-DQ2.5 binds and presents immunogenic gliadin peptides more effectively than HLA-DQ8 and HLA-DQ2.2 (32, 33). Homozygosity of the HLA-DQB1*02 allele, which encodes the beta chain of HLA-DQ2, may impact the number of gliadin-specific T cells that are activated after gluten exposure. Patients with HLA-DQ2 who are homozygous for the HLA-DQB1*02 allele appear more likely to respond to a gluten challenge with increased serum IL-2 and to have higher maximum serum concentrations of IL-2 than other genotypes (24, 34). In turn, this subset of patients may have a slower intestinal recovery rate after gluten challenge (14) and may be more likely to progress to RCD2 (35). These findings support HLA DQB1*02 homozygosity as a determinant of gluten response and a potential prognostic biomarker for predicting the course of disease. Similar findings have not been associated with the gene for the alpha chains of HLA-DQ2.5, or HLA-DQ2.2, or the genes for the alpha or beta chains of HLA-DQ8.

**Table 1:** Biomarkers used in celiac studies.

Biomarker	Biomarker type^a^	Context of use in clinical studies	Sample type (collected)	Assay^b^	Representative clinical studies^c^
HLA-DQ2 and/or HLA-DQ8	Prognostic Diagnostic	Confirm diagnosis	DNA (blood cells or cheek swab)	Molecular assay (13)	(10, 14)
Villous height:crypt depth ratio	Diagnostic Pharmacodynamic Monitoring	Confirm diagnosis Monitor response to therapy	Protein (mucosal biopsy)	IHC (15)	(9, 10)
IEL count	Diagnostic Pharmacodynamic Monitoring	Confirm diagnosis Monitor response to therapy	Protein (mucosal biopsy)	IHC (15)	(9, 10)
Celiac serology TTG-IgA level	Diagnostic Pharmacodynamic Monitoring	Confirm diagnosis Determine if gluten exposure has occurred	Protein (serum)	ELISA (16)	(9, 10, 17)
Number of HLA-DQ2 restricted gluten peptide binding CD4 T cells	Diagnostic Pharmacodynamic Monitoring	Gluten-specific T-cell response	Protein (blood cells)	HLA-tetramer binding measured by flow cytometry (18)	(19, 20)
Production of γIFN in response to *ex vivo* blood cell culture with gluten peptides (number of spot-forming units)	Pharmacodynamic Monitoring	Gluten-specific T-cell response	Protein (blood cells; PBMC)	γIFN ELISpot (21)	(20, 22)
Production of IP-10 in response to *ex vivo* culture with gluten peptides	Pharmacodynamic Monitoring	Gluten-specific T-cell response	Protein (blood cells; PBMC)	IP-10 ELISA (23)	(23)
Change in IL-2 with oral gluten challenge	Diagnostic Pharmacodynamic Monitoring	Gluten-induced immune response	Protein (serum or plasma)	Ultrasensitive ligand binding assays (24)	(20, 25)
Change in gut-homing γ δ T cells and CD8αβ T cells	Pharmacodynamic Monitoring	Gluten-induced immune response	Protein (blood cells; PBMC)	Mass cytometry or flow cytometry (26)	(20, 26)
RCD2 aberrant lymphocytes per total IELs	Diagnostic Pharmacodynamic	Confirm diagnosis Monitor response to therapy	Flow cytometry or IHC	Flow cytometry (27)	(28)
NKp46 positive IELs/100 epithelial cells	Diagnostic	Confirm diagnosis	IHC	(29)	None
Celiac minutes of enteropathy	Diagnostic Pharmacodynamic Monitoring	Extent of villous damage	Optical images	VCE (30)	(20)

IEL, intraepithelial lymphocyte; γ IFN, gamma interferon; IHC, immunohistochemistry; HLA, human leukocyte antigen; PBMC, peripheral blood mononuclear cell; RCD, refractory celiac disease; TTG-IgA, IgA antibodies against tissue transglutaminase; VCE, video capsule endoscopy.

a FDA-NIH Biomarker Working Group. BEST (Biomarkers, EndpointS, and other Tools) Resource [Internet]. Silver Spring (MD): Food and Drug Administration (US); 2016-. Available from: https://www.ncbi.nlm.nih.gov/books/NBK326791/ Co-published by National Institutes of Health (US), Bethesda (MD).

b The listed assays are the most commonly used technologies, and the indicated references provide information on the technical development and/or an example of clinical validation.

c Representative clinical studies are studies where the indicated biomarker was included as part of the protocol and prospectively collected.

The link between HLA-DQB1*02 homozygosity and disease severity or progression to more complicated disease has not been seen in all populations (36, 37). Whether this discrepancy is a biologically relevant observation showing a lack of prognostic power in the number of HLA-DQB1*02 alleles in these populations, or is a result of a small patient number, biased patient selection, or differences in study protocol, requires further investigation.

## Diagnostic Biomarkers and Patient Selection for Clinical Trials

In CeD, diagnostic biomarkers could be used to confirm that an individual has CeD and not another disease, such as irritable bowel syndrome, that clinically mimics symptoms of CeD (38, 39). They could also be used to distinguish between disease subtypes, for example patients with active CeD due to inadvertent gluten exposure versus patients with RCD1, just as current biomarkers allow for differentiation of RCD1 from RCD2 as described later (40). Distinguishing between patient subpopulations is important because some therapies, such as enzymes that degrade gluten, may be effective at blocking intestinal damage in a patient with disease driven by gluten exposure. However, a patient with RCD2, in whom intestinal damage occurs in the absence of gluten exposure, would have continuing pathological changes despite this type of therapy.

Currently, diagnosis of patients with CeD is based on serology, histology, and genetic biomarkers (e.g. HLA-DQ). In patients on a gluten-containing diet, detection of high titers of IgA antibodies against tissue transglutaminase (TTG-IgA) alone may be sufficient for a diagnosis of CeD (16, 41, 42). However, up to 5% of the Western population follow a GFD as a lifestyle choice (43), and, once a patient is on a GFD, antibodies to TTG and deamidated gliadin peptide subside and serology cannot be used for diagnosis. Confirmation of diagnosis in seropositive and seronegative patients is based on histology of duodenal biopsies and HLA typing (42). HLA-DQ2 or HLA-DQ8 expression alone is not sufficient for disease diagnosis because these are common HLA haplotypes, particularly in Western populations. Therefore, as diagnostic biomarkers, HLA-DQ2 or HLA-DQ8 alone have a negative predictive value of near 100%, but have a negligible positive predictive value (44, 45).

For clinical trial design, these biomarkers have been used in combination to help verify CeD diagnosis, for patient stratification and as part of assessment of disease activity (e.g. active disease, well-controlled disease). In the phase II studies for latiglutenase and larazotide, celiac serology was used to estimate gluten exposure and to identify patients likely to have active disease (9, 17). In addition to celiac serology, the latiglutenase and the AMG 714 phase IIa studies used duodenal histology to characterize epithelial damage, based on villous height:crypt depth ratio (Vh:Cd) and intestinal inflammation as assessed by intraepithelial lymphocyte (IEL) counts (10). In the latiglutenase study, patients were selected based on Vh:Cd of ≤ 2 and were stratified based on serology (9). The success or failure of these biomarkers to reduce variability and segregate patients into clinically meaningful and therapeutically important populations is difficult to assess because neither study met its primary endpoint. However, it is notable that in the latiglutenase study, a *post hoc* analysis found that seropositive patients preferentially showed symptomatic relief compared with seronegative patients (46).

The clinical manifestations of CeD vary widely between patients, as does the pathophysiologic response to gluten exposure. This variability presents a challenge to detecting therapeutic response in clinical studies. To directly reduce variability in patient response, gluten challenge has been incorporated into clinical trials (10, 47). By challenging patients with a specific gluten dose regimen, the temporal changes induced by gluten and the impact of therapy can be measured with a variety of disease-relevant biomarkers as described later. For this type of study, it is critical to know that a patient with CeD has the potential for a robust response to gluten. However, patients recruited into these studies are on a GFD for ≥ 6–12 months and are often intentionally selected based on negative celiac serology. Thus, only intestinal damage and HLA are currently widely available for use as indicators of disease status in these patients, and neither of these tests for a functional gliadin immune response.

One recent innovation, the HLA-DQ–gluten tetramer-based diagnostic assay, has been shown to differentiate patients with CeD from healthy controls, and to differentiate patients on a GFD from those who have recently ingested gluten. Overall, this assay is both sensitive and specific for identifying patients with CeD, regardless of diet. The HLA tetramers, major histocompatibility complex class II molecules loaded with immunogenic peptides, used in the assay have to be from the same HLA-DQ haplotype as those in the patient being tested. HLA-DQ2.5, HLA-DQ8, and HLA-DQ2.2 tetramers have been produced by academic groups, suggesting that the majority of patients with CeD could be tested (32, 48, 49). However, the assay takes a substantial amount of blood to perform, HLA-DQ tetramers are not commercially available and it is labor-intensive, suggesting it may not be feasible as a clinical diagnostic tool (50). A second approach exploits the fact that CD4 T-cell clonotypes are long-lived and persist for decades in patients with CeD (51). This assay sequences the rearranged T-cell receptor (TCR) β chain T cells and was shown to imply a diagnosis of CeD, based on TCR sequences common to patients with CeD (e.g. public sequences). The proof of principle was done using lamina propria T cells enriched with gluten-specific CD4 T cells; however, the ultimate goal of this approach will be to do the same assay in blood (52). The utility of these assays to confirm disease status as part of a clinical trial has yet to be tested.

Diagnosis of CeD subtype (uncontrolled CeD, RCD1, RCD2) is also key for the development of therapies, because the mechanisms driving each disease subtype may differ and, particularly for RCD2, which has a high mortality, the benefit–risk profile is quite different. RCD1 cannot be distinguished from gluten-induced active CeD *via* biomarkers. However, RCD2 can be identified based on biomarkers. The RCD2 IEL population is distinct from the polyclonal CD3+, CD8+ IELs associated with inflammation and damage after gluten challenge. RCD2 IELs are clonal TCR rearrangements and are positive for NKp46, tend to be CD8- and do not express surface CD3 or TCRs (29, 40). In a single phase IIa study, the safety and efficacy of anti-IL-15 (AMG 714) were tested in patients with RCD2. Patients were selected based on the percentage of aberrant IELs/100 total CD45+ IELs by flow cytometric analysis or > 50% aberrant IELs as measured by immunohistochemistry. This study did not meet the primary endpoint, reduction of aberrant intraepithelial lymphocytes from baseline measured at 12 weeks (28).

## Pharmacodynamic Biomarkers and Measuring Therapeutic Intervention in CeD

Pharmacodynamic (PD) biomarkers measure the impact of therapeutic intervention on a biological process. In the development of therapies for CeD, PD biomarkers could be used to evaluate therapeutic target engagement, gluten exposure or measure clinically meaningful endpoints, such as change in gluten-specific T cells and resolution of intestinal damage. Some PD markers can be tested serially to monitor drug-mediated changes over time and durability of therapeutic-induced responses to help build a rationale for a dosing regimen. A caveat for these PD biomarkers is that serial collection should have minimal impact on patient comfort or safety, thus, less invasive blood-based or imaging biomarkers are favored over duodenal biopsies.

PD biomarkers used in previous CeD clinical trials quantitatively measured changes in small intestine epithelial damage and inflammation by histology and evaluated immune response to gluten exposure by serology (9, 10). As a PD marker, histology has the advantage of measuring changes that are directly related to CeD processes, the influx of T cells into the epithelium, and the subsequent destruction of mucosal epithelium. Moving from the use of a subjective scoring system (such as Marsh–Oberhuber grade) to a quantitative evaluation of intestinal changes (e.g. measuring Vh:Cd ratio and IEL numbers), provides the sensitivity to detect relatively small, but clinically significant damage (15, 53). Vh:Cd is currently the standard for mucosal assessment in CeD clinical trials and is more reliable and responsive than traditional subjective histological measures. However, reliance on Vh:Cd has several limitations, including: mucosal biopsies are invasive and unsuited for serial testing, duodenal biopsy provides only a small representation of the entire disease area, expertise and significant time is needed to properly orient tissue sections, and Vh:Cd does not include a measure of lymphocytosis (34). Video capsule endoscopy (VCE) avoids these issues. It is less invasive than a duodenal biopsy and can be used as a method to monitor therapeutic impact while evaluating a much larger portion of the small intestine. VCE is unable to directly measure cellular changes, but rather records macroscopic changes in tissue (20, 30).

Anti-TTG antibodies have been used as a PD biomarker to understand immune response to gluten challenge (19, 20, 54) and have been incorporated into clinical trials for this purpose (9, 10). These antibodies are not considered by most to be pathogenic in the intestinal damage seen in CeD, but may be a contributor to extraintestinal manifestations, such as dermatitis herpetiformis or central nervous system lesions (55–57). Antibody response requires repeated gluten exposure, takes at least 2 weeks to appear after initial gluten challenge, and is still high 3–4 weeks after the last gluten exposure (20, 54). Although anti-TTG antibody measurement is useful for CeD diagnosis, it is certainly not a dynamic biomarker. In comparison, newer cytokine and cellular PD biomarkers are more responsive to gluten, with changes seen in days or hours after gluten challenge, quickly dropping to pre-gluten challenge levels. After a single gluten dose in patients on a GFD, levels of several inflammatory cytokines increase (58). IL-2 is one of the most consistently upregulated cytokines in patients and peaks 4 hours after gluten challenge, becoming undetectable in most patients by 6 days after initial gluten exposure (20). The presence of IL-2 in patients correlated with CeD symptoms, and no changes in IL-2 were seen in healthy participants with gluten challenge (58, 59).

Gluten-specific CD4 T cells are released into the blood 6 days after the start of a gluten challenge (20, 22). Gluten-specific T cells can be induced by *ex vivo* antigen challenge with gluten peptides and quantified by γIFN ELISpot, IP-10 ELISA or visualized by flow cytometry using HLA-DQ2 tetramers in combination with CD38 expression (60). The results of these assays correlate well with each other (22, 23, 50). As biomarkers for use in clinical trials, they have some pragmatic challenges: they require viable blood cells, reagents that are not commercially available, and large volumes of blood and CD4+ T-cell enrichment (tetramer assay only). However, the role of these gluten-specific CD4 T cells in CeD is clear, and a reduction in these cells would be highly suggestive of a disease-modifying effect.

Along with gluten-specific T cells that arise after gluten challenge, gut-homing CD8αβ T cells and γδ T cells that co-express CD103 and the activation antigen, CD38, also increase 6 days after the start of a gluten challenge. Although the role of these cells in CeD is less well understood, the gut-homing CD8 T cells are phenotypically similar to IELs found in patients with active CeD (26). The advantage of tracking these cells as a PD biomarker of active disease is that they are more plentiful in the blood and do not require pre-enrichment or cell culture prior to staining.

## Discussion

Translational medicine and biomarkers are becoming integral components of clinical development, contributing to trials by: 1) supporting dose and dose regimen selections for new therapeutic modalities that preclude traditional pharmacokinetic measures; 2) confirming unique therapeutic mechanisms of action; 3) providing proof of concept earlier in development; and 4) showing therapeutic efficacy in trials that require fewer patients. Because the etiology of CeD is better understood than that of most chronic inflammatory diseases, it has been possible to design biomarker assays that allow quantification of the earliest changes induced by gluten ingestion, tracking of the adaptive immune response, and evaluation of tissue inflammation and damage. However, biomarkers have some limitations in a real-world setting, and are only one approach to understanding disease progression and therapeutic efficacy (61). With advances in technology and the discovery of new biomarkers, it is possible that, in future studies, patient selection can be based on specific disease subtypes, or on prognosis, identifying the patient subpopulation most appropriate for the benefit–risk profile of a given therapy. From early clinical studies, pharmacokinetic data and data from PD markers can be combined to model the therapeutic dose response and gain a deeper understanding of the therapeutic mechanism of action.

## Author Contributions

GS, JM, and DL were responsible for the conceptualization of the manuscript. GS wrote the original draft. All authors reviewed and edited the manuscript. All authors contributed to the article and approved the submitted version.

## Funding 

This study was sponsored by Takeda Pharmaceutical Company Ltd. Editorial support was provided by Oxford PharmaGenesis, Oxford, UK and was funded by Takeda Pharmaceutical Company Ltd.

## Conflict of Interest

The authors declare that this study was funded by Takeda Pharmaceutical Company Ltd. All authors were employees of Takeda Pharmaceuticals Inc. Co., and were responsible for the content, collection, analysis, interpretation of data, preparation of this article and the decision to submit it for publication.

## Abbreviations

CeD, celiac disease; GFD, gluten-free diet; IEL, intraepithelial lymphocyte; γIFN, gamma interferon; IHC, immunohistochemistry; HLA, human leukocyte antigen; PBMC, peripheral blood mononuclear cell; PD, pharmacodynamic; RCD, refractory celiac disease; RCD1, RCD type 1; RCD2, RCD type 2; TCR, T-cell receptor; TTG-IgA, IgA antibodies against tissue transglutaminase; VCE, video capsule endoscopy; Vh:Cd, villous height:crypt depth.
